# Overexpression of *RAD51* Enables PCR-Based Gene Targeting in Lager Yeast

**DOI:** 10.3390/microorganisms7070192

**Published:** 2019-07-05

**Authors:** Beatrice Bernardi, Yeseren Kayacan, Madina Akan, Jürgen Wendland

**Affiliations:** 1 Department of Microbiology and Biochemistry, Hochschule Geisenheim University, von-Lade Strasse 1, D-65366 Geisenheim, Germany; 2 Research Group of Microbiology (MICR)—Functional Yeast Genomics, Vrije Universiteit Brussel, BE-1050 Brussels, Belgium

**Keywords:** homologous recombination, gene function analysis, hybrid yeast, fermentation

## Abstract

Lager beer fermentations rely on specific polyploid hybrids between *Saccharomyces cerevisiae* and *Saccharomyces eubayanus* falling into the two groups of *S. carlsbergensis/Saaz*-type and *S. pastorianus/Frohberg*-type. These strains provide a terroir to lager beer as they have long traditional associations and local selection histories with specific breweries. Lager yeasts share, based on their common origin, several phenotypes. One of them is low transformability, hampering the gene function analyses required for proof-of-concept strain improvements. PCR-based gene targeting is a standard tool for manipulating *S. cerevisiae* and other ascomycetes. However, low transformability paired with the low efficiency of homologous recombination practically disable targeted gene function analyses in lager yeast strains. For genetic manipulations in lager yeasts, we employed a yeast transformation protocol based on lithium-acetate/PEG incubation combined with electroporation. We first introduced freely replicating *CEN/ARS* plasmids carrying *ScRAD51* driven by a strong heterologous promoter into lager yeast. *RAD51* overexpression in the *Weihenstephan* 34/70 lager yeast was necessary and sufficient in our hands for gene targeting using short-flanking homology regions of 50 bp added to a selection marker by PCR. We successfully targeted two independent loci, *ScADE2/YOR128C* and *ScHSP104/YLL026W*, and confirmed correct integration by diagnostic PCR. With these modifications, genetic alterations of lager yeasts can be achieved efficiently and the *RAD51*-containing episomal plasmid can be removed after successful strain construction.

## 1. Introduction

Lager beer is the most popular alcoholic beverage worldwide. It is distinguished from top-fermented ale beers by the use of dedicated bottom fermenting lager yeasts. Pure culture lager yeasts were introduced in the late 19th century and the first lager yeast strain became known as *Saccharomyces carlsbergensis* [1,2]. Lager yeasts are particularly suited for cold fermentations below 15 °C, providing a specific and clean taste [3]. Lager yeast strains are hybrids of two *Saccharomyces* species, namely *S. cerevisiae* and *S. eubayanus* [4,5,6,7,8]. The *S. cerevisiae* parent is related to ale yeasts while the *S. eubayanus* parent has recently been isolated from South America and Asia [9,10]. The low temperature fermentation capabilities of lager yeast hybrids may have originated from the parental *S. eubayanus* mitochondrial DNA that is invariably present in today’s lager yeasts [11].

Genetic manipulations in lager yeast are hampered by the allopolyploid nature of these hybrids but also by their low transformation and homologous recombination (HR) efficiency [12]. This is demonstrated by the very few reports on targeted gene alterations in lager yeast [13,14,15]. These shortcomings of lager yeasts are in sharp contrast to the high efficiency and ease with which *S. cerevisiae* can be manipulated [16]. A large array of modules for PCR-based gene targeting have been developed for *S. cerevisiae* and the gene deletion collection was one of its outputs that spurred large scale functional and synthetic genetic array analyses [17,18,19,20,21,22]. PCR-based gene targeting tools were also developed for other ascomycetous fungi with efficient homologous recombination machineries, including *Ashbya gossypii, Candida albicans* and *Schizosaccharomyces pombe* [23,24,25,26,27,28,29].

Deletion of Ku70 or Ku80 of the non-homologous end joining (NHEJ) pathway has been shown to be a very useful tool to improve gene targeting in a large variety of fungi, including e.g., *Kluyveromyces lactis* and *Yarrowia lipolytica* [30,31,32]. The Ku70 deletion approach has been particularly successful in filamentous ascomycetes in which non-homologous end joining is otherwise predominant, e.g., in *Neurospora crassa* or *Zymoseptoria tritici* [33,34]. However, in *Cryptococcus neoformans* it was found that deletion of Ku70 also alters virulence, which then requires sexual crosses to reinstate wildtype Ku70 in a mutant background [35].

An alternative to impairing NHEJ is improving HR efficiency. Overexpression of *RAD51,* coding for a protein involved in the recombinational repair of DNA-double strand breaks, in *S. cerevisiae* enhances gene repair, a feature conserved in human cells that can also be achieved by using *RecA* from *Escherichia coli* [36,37,38]. We thought to employ the apparently highly conserved nature of HR improvement upon *RAD51* overexpression in lager yeast. Here we show that plasmid-based overexpression of *ScRAD51* under the control of a strong heterologous promoter, allows for simplified targeted gene function analyses in lager yeast. This genetic improvement of lager yeast transformation was combined with a lithium acetate/electroporation transformation protocol similar as has previously been described for *S. cerevisiae* and *S. pombe* [39,40]. Together, these improvements should aid proof-of-concept analyses in lager yeasts prior to embarking on laborious mutagenesis and selection schemes for strains not genetically modified (non-GM) used in the production of fermented beverages. Additionally, this provides a tool to investigate hybrid biology at the molecular level.

## 2. Material and Methods

### 2.1. Strains and Culture Conditions

Yeast strains, used and generated (see Table 1), were grown in YPD (1% yeast extract, 2% peptone, 2% glucose) at 30 °C. Solid media were prepared by adding 2% agar and YPD plates were supplemented with either hygromycin or G418/geneticin. For lager yeasts the antibiotic concentrations used were 100 µg/mL, while *S. cerevisiae* lab strains were selected at 200 µg/mL final concentration. Minimal media lacking leucine contained 6.7 g/L yeast nitrogen base with NH_4_SO_4_ and without amino acids and 0.69 g/L completed synthetic medium leucine drop-out mixture. Plasmids were propagated in *Escherichia coli* DH5alpha in 2xYT (1.6 % tryptone, 1 % yeast extract, 0.5 % NaCl) containing 100 µg/mL ampicillin.

### 2.2. Plasmid Design and Constructions

The plasmids used in this study are listed in Table 2. The primers required for plasmid constructions or for PCR-based gene targeting are listed in Table 3. Plasmid DNAs were amplified in *E. coli* and prepared using a Plasmid Midi DNA Purification Kit (Genaxxon, Ulm, Germany). PCR fragments were cloned into pGEM (Promega, Madison, USA). The *ScRAD51/YER095W* open reading frame was amplified from BY4741 genomic DNA using primers 260 and 261 and the *Arthroascus* (=*Saccharomycopsis*) *schoenii TEF1* promoter was amplified from *A. schoenii* genomic DNA using primers 106 and 107, and both were cloned into pGEM. To place *LacZ* under the control of the *AsTEF1*-promoter, *AsTEF1p* was amplified form pGEM-AsTEF1p (E026) with primers 108/109, which added 45 bp of flanking homology region to E025-pRS417-AgTEFp-LacZ (here *LacZ* is under the control of the *Ashbya gossypii TEF*-promoter). The *AgTEF*-promoter was removed from E025 by restriction digestion with *Kpn*I/*Xho*I, and the vector and *AsTEF1p* were cotransformed into yeast to combine them via in vivo recombination, yielding plasmid E065. All restriction enzymes were obtained from Thermo Fisher (Asse, Belgium). All primers were obtained from Sigma Aldrich (Overijse, Belgium).

E150/pRAD51 (*GEN3*) was constructed by in vivo recombination of E065 vector backbone with fragments carrying *ScRAD51*-ORF and *HYG3* marker. To this end, E065 was linearized by digesting with *Eco*RV/*Sac*I, which removed most of the *LacZ*-ORF, and then the band was gel purified. Flanking adaptor regions, to guide in vivo recombination, were added to *ScRAD51*-ORF and *HYG3* marker fragments using primers 258/259 and 253/254, respectively. Then BY4741 was transformed with the two PCR products and the cut vector. In order to use a G418 resistance marker for PCR-based gene targeting in lager yeast, the *GEN3* marker in E150 was replaced by *LEU2* in a PCR-based gene targeting approach using BY4741 as a host. To this end, *ScLEU2* was amplified from pRS415 with primer 344/345 by PCR and used for transformation, generating E160.

Novel synthetic marker genes *YES1* and *YES2* were also constructed using in vivo recombination in *S. cerevisiae* using plasmid pRS415 as the vector backbone. *YES1* and *YES2* contain the *AsTEF1*-promoter that controls kanamycin or hygromycin resistance gene-ORFs, respectively. For construction, pRS415 was linearized with *Sma*I, the *AsTEF1p* was amplified from E026 using primers 226/227, the *kanR*-ORF was amplified with primers 228/229 and the *hygR*-ORF with primers 230/231 to add suitable adaptors for homologous recombination. Vector, promoter and resistance gene ORFs were then cotransformed in *S. cerevisiae* and the resulting transformants were selected on G418 or hygromycin, respectively. All plasmids obtained by in vivo recombination in *S. cerevisiae* were verified and shuttled into *E. coli* for propagation. *A. schoenii* promoters were used as heterologous promoters in lager yeast and *S. cerevisiae*. 

*HYG3* is a synthetic selectable marker that consists of the 694 bp *A. schoenii PGK1* promoter, the 1026 bp hygromycin resistance gene ORF and the *Candida albicans URA3* terminator, which was synthesized by GenScript (GenScript, Piscataway, NJ, USA). Promoter sequences were derived from the *A. schoenii* CBS 7425 genome, which can be accessed via GenBank under accession JNFU00000000 CBS 7425 [43].

### 2.3. Yeast Transformation

For the transformation of *S. cerevisiae,* the lithium acetate/single strand DNA/polyethylene glycol 4000 protocol was used as described [44]. For lager yeast transformation, we adapted a protocol from Thompson et al. [39]. WS34/70 was grown overnight to stationary phase in 50 mL YPD. Then the culture was diluted to OD_600_ = 0.3 in 100 mL fresh YPD. Cells were harvested at mid-log growth with OD_600_ = 0.7–0.8 (after approximately 3 h of incubation). The culture was centrifuged and washed once with sterile water and resuspended in 25 mL of 0.1 M lithium acetate/10 mM dithiothreitol/10 mM TE solution (Tris HCl: EDTA = 10:1) and incubated for 1 h at room temperature. The cells were washed in 25 mL ice-cold distilled sterile water twice and once with 10 ml of ice-cold 1 M sorbitol, then resuspended in 100 µL ice-cold sorbitol. A 100 µL aliquot of the cell suspension was used for transformation. Transforming DNA (15 µL) was mixed with the cells, incubated for 5 min on ice and electroporated with 1.8 kV in 0.2 cm cuvettes. Then, cells were resuspended in 1 ml cold sorbitol and transferred to a tube with 300 µL YPD. This suspension was incubated for 3 h at 30 °C prior to plating on selective plates that did not contain sorbitol.

### 2.4. Verification of Lager Yeast Transformants

The *Weihenstephan* strain WS34/70 was used for gene targeting experiments. Target genes were derived from the *S. cerevisiae* parental subgenome. Sequences for verification primers (and also for the S1 and S2 primers) were deduced from the genome sequence that is available at DDBJ/EMBL/GenBank under the accession AZAA00000000. PCR-based gene targeting and diagnostic PCR were performed as described [45].

## 3. Results

### 3.1. Design and Testing of Synthetic Marker and Reporter Genes

**Figure 1:**
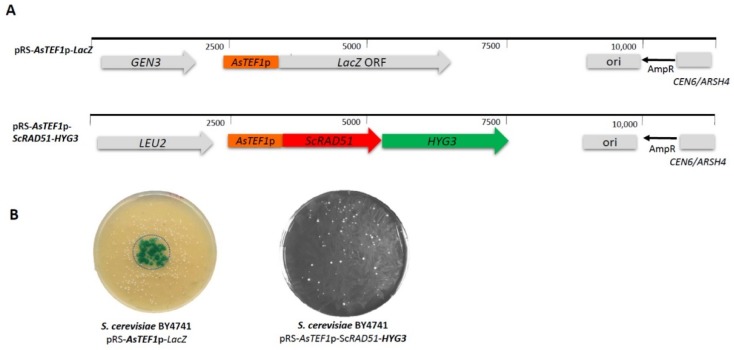
Generating heterologous synthetic marker and reporter genes. (**A**) Linear maps of pRS-*AsTEF1*p-*LacZ* and pRS-*AsTEF1*p-*ScRAD51*-*HYG3* plasmids. (**B**) The *AsTEF1*p-LacZ reporter gene and the *HYG3* resistance marker were tested in *Saccharomyces cerevisiae* BY4741. The presence of an active β-galactosidase was detected by adding X-Gal to the centre of the transformation plate (left panel, circle indicates the area of X-Gal application). The presence and function of the *HYG3* marker was tested by transforming *S. cerevisiae* with pRS-*AsTEF1*p-*ScRAD51*-*HYG3* and selecting the transformants in the presence of 100 µg/mL of hygromycin (right).

We were interested in developing novel synthetic marker and reporter genes for dual use in non-conventional yeasts, including lager yeasts and *S. cerevisiae*. To this end, we tested heterologous promoters derived from *Arthroascus schoenii* (*Saccharomycopsis schoenii*). *Saccharomycopsis* species are non-conventional yeasts used in diverse brewing settings, e.g., to generate nuruk [46,47]. We recently obtained several *Saccharomycopsis* genome sequences including that of *A. schoenii* [43]. Here we fused the *AsTEF1* promoter with the *LacZ* reporter gene derived from *Streptococcus thermophilus* [48]. Additionally, we generated a completely new synthetic marker gene based on the *AsPGK1* promoter controlling a hygromycin resistance gene ORF. Both genes were introduced into BY4741 on episomal *CEN6/ARSH4* plasmids (Figure 1). The functionality of both the synthetic marker and the reporter were demonstrated in vivo in *S. cerevisiae*: *HYG3* was functional as we obtained hygromycin-resistant transformants that carried the plasmids, and the *LacZ* reporter was active as transformants were able to convert colorless X-Gal into a blue dye (Figure 1B). This indicates that both *A. schoenii* promoters derived from *AsTEF1* and *AsPGK1* were functional in the heterologous host *S. cerevisiae* and we thus went on to employ these genes in lager yeast.

### 3.2. Transformation of Lager Yeast

Previous analyses already indicated that lager yeasts’ transformation efficiency is drastically lower than that of *S. cerevisiae.* In *S. cerevisiae* lithium acetate/ss DNA/PEG protocols are commonly used [44]. We compared this standard protocol with modified versions, which were developed to deal with yeast strains that are non-responsive to standard lithium acetate or electroporation protocols [39,40]. Our modified protocol included parts of both protocols: a lithium acetate treatment (without DMSO) and an electroporation step (Li-Ac/Ep). To allow time for the expression of the antibiotic resistance genes used in this study, we incubated the cell suspensions after electroporation in an osmotically stabilized medium before plating on selective plates. To compare both protocols, we employed a freely replicating plasmid (E160) with *HYG3* as the selectable marker.

**Figure 2:**
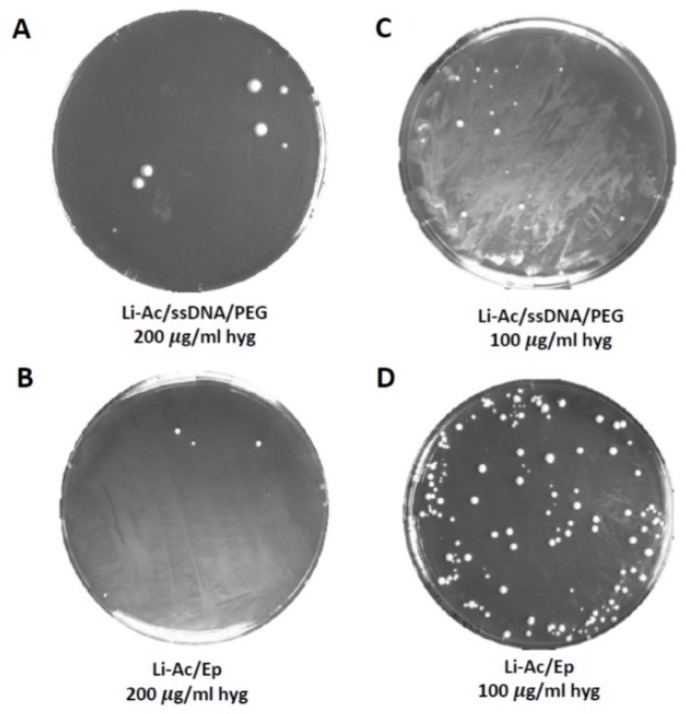
Comparison of two transformation methods in the *Weihenstephan* lager yeast strain WS 34/70. (**A**,**C**) A lithium-acetate/single strand DNA/polyethylene glycol (Li-Ac/ssDNA/PEG) and (**B**,**D**) a LiAc/PEG incubation combined with electroporation (Li-Ac/Ep) were used with two different antibiotic concentrations for the selection of transformants. The transformation of WS 34/70 with a freely replicative plasmid carrying a hygromycin resistance marker (pRS-*AsTEF1*p-*ScRAD51*-*HYG3*) yielded few transformants when using the Li-ac/ssDNA/PEG method (**A**,**C**). By contrast, the Li-Ac/Ep method together with antibiotic selection at 100 µg/mL final concentration (**D**), resulted in a higher transformation efficiency compared to the other combinations (**A**–**C**).

In our hands, the lithium acetate/ss DNA/PEG protocol (with DMSO) yielded only a few transformants per µg of DNA, demonstrating the poor transformation efficiency of lager yeast with this method, even after lowering the temperature of the heat shock step at 40 °C, which we tested as lager yeasts are known to be temperature sensitive and do not grow at elevated temperatures above 34 °C. The Li-Ac/Ep protocol, however, resulted in much higher transformation efficiencies (1–2 orders of magnitude, Figure 2).

### 3.3. PCR-Based Gene Targeting is Enhanced by RAD51 Overexpression

**Figure 3:**
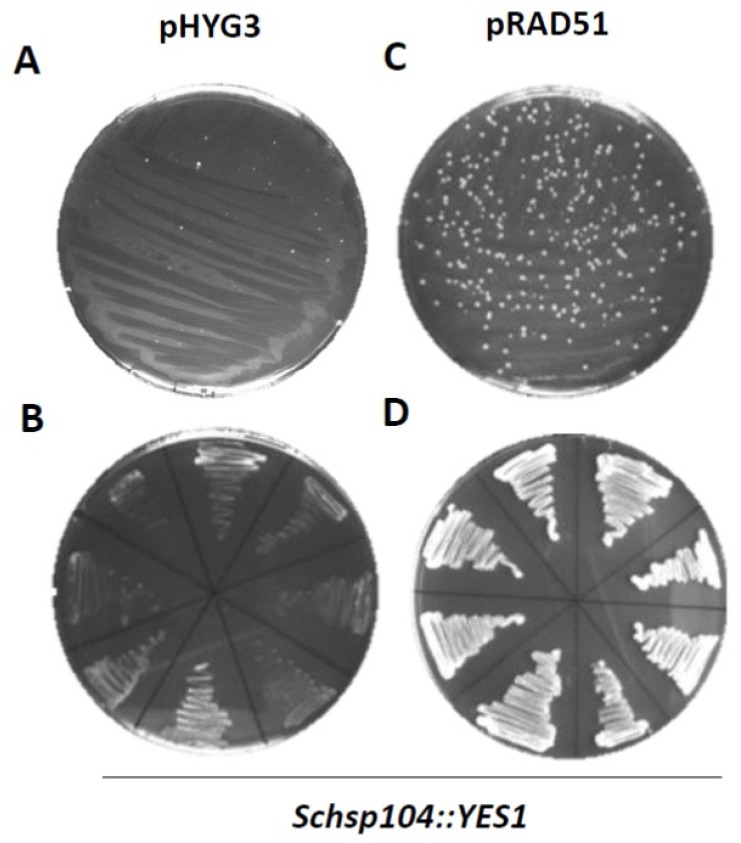
PCR-based gene targeting in lager yeast. The *Weihenstephan* strain WS34/70 carrying either an empty vector (pHYG3; **A**) or a *RAD51* plasmid (pRAD51, **C**) was transformed with disruption cassettes containing the *YES1* marker harboring 50 bp of flanking homology regions for targeting to the *ScHSP104* locus. We used a Li-Ac/Ep transformation protocol and selected transformants on YPD plates supplemented with hygromycin and G418 (100 µg/mL final concentration each). Restreaking of putative transformant colonies on new selective plates indicated stable transformants were only obtained in the lager yeast strain overexpressing pRAD51 (**B**,**D**).

Plasmid transformation is regularly far more efficient than integrative transformation. PCR-based gene targeting approaches are convenient because in one PCR reaction short-flanking homology regions can be added to a selection marker. We used standard S1 and S2 primers that added 50 bases of flanking homology region. However, in our hands, we failed to obtain stable lager yeast transformants with these PCR-based disruption cassettes, even when using the Li-Ac/Epo protocol (Figure 3A). After prolonged incubation on selective plates (>2d) small colonies may appear. However, these colonies did not continue to grow upon restreaking on new selective plates or in selective liquid media and represent background growth (Figure 3B). In contrast, the transformation of a *Weihenstephan* lager yeast strain that harbors a plasmid-encoded *ScRAD51* expressed from a strong *TEF* promoter yielded a large number of transformants on primary selective plates using disruption cassettes for *ScHSP104* (Figure 3C). A randomly selected set of colonies continued to grow upon restreaking and also grew well in liquid YPD supplemented with hygromycin (Figure 3D and data not shown).

**Figure 4:**
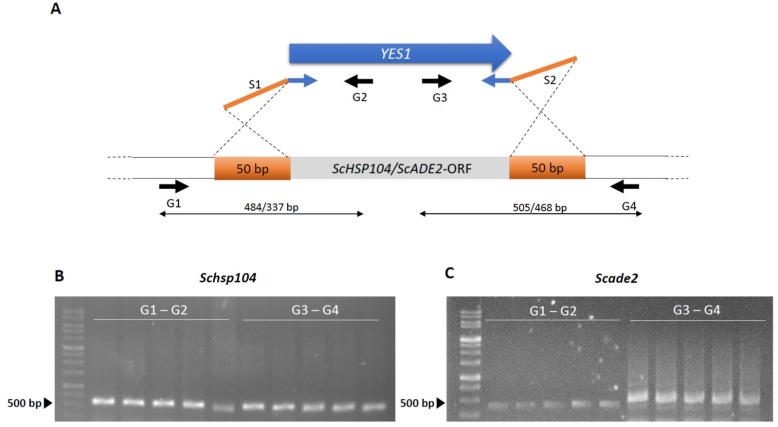
Diagnostic PCR for verification of correct marker integration. (**A**) Schematic presentation of PCR-based gene targeting of *YES1* amplified with gene specific S1/S2-primers adding 50 bp of target homology region (orange bars) to *ScHSP104* or *ScADE2*, respectively. Diagnostic G1/G2 and G3/G4 primers were used for verification of the correct insertion of *YES1* and removal of the *Sc HSP104* (**B**) and Sc *ADE2* locus (**C**) ORFs.

Verification of these transformants that grew upon restreaking and growth in liquid culture was done by standard diagnostic PCR, amplifying the novel joints at the borders of marker integration [45]. This confirmed targeted gene disruption (Figure 4). Overall, between 60–75% of primary transformants could be cultivated further in a liquid culture, and all for all of those diagnostic PCRs indicated correct integration of the marker cassette at the target locus (*n* > 20). However, we did not obtain any transformants in the same strain without the overexpression of *ScRAD51*, demonstrating the usefulness of this approach. To verify that the marker integration and PCR-based gene targeting success was not solely locus dependent, we targeted a second gene, *ScADE2*, in the same manner. Transformation efficiencies were found to be similar for both loci and diagnostic PCR was successfully employed for the verification of the deletion of an *ADE2* allele (Figure 4C).

## 4. Discussion

Lager yeasts are the workhorses of the beer industry. Detailed strain characterizations and molecular genetics-assisted yeast breeding, however, are hampered by the lack of proof-of-concept technologies. A major obstacle in lager yeast research is the surprisingly inefficient gene targeting in lager yeast. Thus, there are only a few reports of the successful molecular genetic manipulation of lager yeast relying on HR [13,14,15]. There are other issues beyond the mere technical difficulties. Primarily, these are complicated by the hybrid nature of lager yeast. Lager yeasts are allopolyploid hybrids between *S. cerevisiae* and *S. eubayanus* parents [9]. Group I/Saaz strains are triploid, while Group II/Frohberg strains, to which *Weihenstephan* 34/70 belongs, are tetraploid [2]. Additionally, aneuploidies could further change allele frequencies, complicating gene knockout experiments. Secondly, due to the hybrid nature, sporulation is severely crippled due to failure to proceed through meiotic divisions, resulting in hybrid sterility, which is more pronounced in triploid Group I strains than in tetraploid Group II strains [49,50]. Thirdly, even though lager yeasts are the workhorses of the beer industry, there is a reluctance to employ genetically modified yeast strains, a notion that has rather been strengthened over recent years.

In addition to their industrial importance, lager yeast hybrids present excellent model systems to study hybrid biology, including hybrid vigor, hybrid sterility, adaptive evolution and the analysis of hybrid protein complexes [51,52]. Major advances in hybrid yeast breeding resolved the F1-sterility problem represented in lager yeast [53].

Our study indicates that *RAD51* overexpression in lager yeast opens the tool-box for all genetic manipulations previously only available in *S. cerevisiae*. Although transformation efficiencies in lager yeasts are far lower than in *S. cerevisiae,* rational strain design based on targeted gene replacements has become feasible. We have successfully employed this strategy already to other loci, suggesting that potential locus dependent variations in gene targeting are not inhibitory to successful gene targeting. Furthermore, by selecting specific homology regions, even closely related alleles of the *S. cerevisiae* and *S. eubayanus* parental genomes can be distinguished. The overexpression of *RAD51* has been shown to improve HR in other systems, so it may also be advantageous in other non-conventional yeasts in which molecular genetic studies are hampered by the preferential ectopic integration of gene targeting cassettes.

Two additional advances to improve HR in resilient strains in recent years include the use of Ku70 mutant strains and the establishment of CRISPR/Cas9 methodologies [12]. Deletion of Ku70 inactivates the NHEJ pathway, thus favouring HR and targeted gene alterations [33]. However, in *S. cerevisiae*, the deletion of Ku70 has been shown to generate defects in telomere maintenance and cell cycle regulation [54,55]. Thus, this may not be favorable in lager yeast as breeding efforts to restore a wildtype Ku70 status after genetic engineering are also quite laborious.

Recent reports established CRISPR/Cas9 in lager yeast, making this a promising tool for strain engineering [12,56]. CRISPR/Cas9 will be particularly useful for simultaneous alterations of multiple alleles in a single step, even more so when the number of alleles may vary due to aneuploidies. In a recent report, simultaneous single and double deletions of *SeATF1* and *SeATF2* were performed in lager yeast, demonstrating the power of this tool [12]. It will be interesting to see if a combined approach, overexpressing *RAD51* and the use of CRISPR/Cas9, will further improve gene replacement efficiencies.

## Figures and Tables

**Table 1:** Strains used in this study.

Strain Number	Feature/Genotype	Source
B003	*Saccharomyces cerevisiae* BY4741 *MATa his3*Δ1; *leu2*Δ0; *met15*Δ0; *ura3*Δ0	Euroscarf
B237	*Weihenstephan WS34/70 lager yeast*	Lab collection
B256	*WS34/70; pRAD51 (HYG3, CEN6/ARSH4)*	This study
B257	*WS34/70; pHYG3 (CEN6/ARSH4)*	This study
G001	*WS34/70; pRAD51; ade2::YES1*	This study
G002	*WS34/70; pRAD51; hsp104::YES1*	This study

**Table 2:** Plasmids used in this study.

Strain Number	Feature/Genotype	Source
E008	pRS415	[41]
E025	pRS417-AgTEF1p-LacZ-GEN3	[42]
E026	pGEM-AsTEF1p	This study
E054	pUC57-HYG3	GenScript
E065	pRS417-AsTEF1p-LacZ	This study
E066	pYES1	This study
E068	pYES2	This study
E088	pGEM-YES1	This study
E120	pGEM-ScRAD51	This study
E150	pRS-AsTEF1p-ScRAD51-HYG3-GEN3	This study
E160	pRS-AsoTEF1p-ScRAD51-HYG3-LEU2	This study

**Table 3:** Primers used in this study.

Primer Number	Primer Name	Sequence 5′→3′ *
106	5′-AsTEF1p	GTCCAGAATAACATCAAATC
107	3′-AsTEF1p	CTATAAAAAATGTTAGTATGGAG
108	5′-AsTEFp-pRS	CGCCAGGGTTTTCCCAGTCACGACGTTGTAAAACGACGGCCAGTGCTCGAGTCCAGAATAACATCAAATC
109	3′-AsTEFp-lacZ	CAATCTTTGGATCGTTTAAATAAGTTTGAATTTTTTCAGTCATGTTCTATAAAAAATGTTAGTATGGAG
226	P3-pRS415-AsTEF1p	TGTAAAACGACGGCCAGTGAGCGCGCGTAATACGACTCACTATAGGAAGCTTCGTACGCTGCAGGTCGGATCCCCCGGGGGCGCGCCGTCCAGAATAACATCAAATC
227	P4-AsTEF1p-kanR	GTTGGAGTTCAAACGTGGTCTGGAAACGTGAGTCTTTTCCTTACCCTATAAAAAATGTTAGTATGGAG
228	P5-kan-ORF	GGTAAGGAAAAGACTCACGTTTCCA
229	P6-kanR-pRS415	GGAAACAGCTATGACCATGATTACGCCAAGCGCGCAATTAACCCTTCTGATATCATCGATGAATTCGAGCTCGTTTAAACATTGGTAATAG
230	P7-5′-HYG3	CTGACTTTTGTCTTGTTATGGACTCCATACTAACATTTTTTATAGAAAAAACCAGAATTGACTGCTACTTC
231	P8-3′-HYG3	CTGATATCATCGATGAATTCGAGCTCGTTTAAACATTGGTAATAGGACCACCTTTGATTGTAAATAG
253	5-HYG3+AD	GGCGCGCCAGATCTAGCCTCCTCAGAGAAAATTGCACAAAAAAAAGGAAGCTTCGTACGCTGCAGGTC
254	3-HYG3+AD	TTACGCCAAGCGCGCAATTAACCCTCACTAAAGGGAACAAAAGCTGACCACCTTTGATTGTAAATAG
258	5-ScRAD51+AD	CTGACTTTTGTCTTGTTATGGACTCCATACTAACATTTTTTATAGTCTCAAGTTCAAGAACAACATATATCAG
259	3-ScRAD51+AD	CTTTTTTTTGTGCAATTTTCTCTGAGGAGGCTAGATCTGGCGCGCCGAAAAATACATATATTTCATGGGTGACAG
260	5-ScRAD51	TCTCAAGTTCAAGAACAACATATATCAG
261	3-ScRAD51	GAAAAATACATATATTTCATGGGTGACAG
344	S1-LEU2	GGGGCTGGCTTAACTATGCGGCATCAGAGCAGATTGTACTGAGAGTAAAGTGCAATTCTTTTTCC
345	S2-LEU2	CTTGTTCCAAACTGGAACAACACTCAACCCTATCTCGGTCTATTCGGTCGAGGAGAACTTC
355	G2-YES1	GAATGAATCTACTGGTTTGG
356	G3-YES1	GTGTCGGTATCGCAGAC
369	S1-ADE2	CCTACTATAACATTCAAGAAAAACAAGAAAACCGGACAAAACAATCAAGTGTCCAGAATAACATCAAATC
371	S2-ADE2	TATATCATTTTATATTATTTGCTGTGCAAGTATATCAATAAACTTATATATAATAAATTATTTTTATTGTTG
373	G1-ADE2	GACTCTTGTTGCAGGGCT
389	G4-ADE2	GTGATGCATTGAGCCGCC
390	S1-HSP104	TATATTACTGATTCTTGTTCGAAAGTTTTTAAAAATCACACTATATTAAAGTCCAGAATAACATCAAATC
391	S2-HSP104	AACAAAGAAAAAAGAAATCAACTACACGTACCATAAAATATACAGAATATTAATAAATTATTTTTATTGTTG
392	G1-HSP104	CCCGTATTCTAATAATGGACC
393	G4-HSP104	CAAACTTATGCAACCTGCCAG

* Underlined sequences correspond to restriction sites.
